# C. elegans Germ Cells Switch between Distinct Modes of Double-Strand Break Repair During Meiotic Prophase Progression

**DOI:** 10.1371/journal.pgen.0030191

**Published:** 2007-11-02

**Authors:** Michiko Hayashi, Gregory M Chin, Anne M Villeneuve

**Affiliations:** 1 Department of Developmental Biology, Stanford University School of Medicine, Stanford, California, United States of America; 2 Department of Genetics, Stanford University School of Medicine, Stanford, California, United States of America; Stowers Institute for Medical Research, United States of America

## Abstract

Chromosome inheritance during sexual reproduction relies on deliberate induction of double-strand DNA breaks (DSBs) and repair of a subset of these breaks as interhomolog crossovers (COs). Here we provide a direct demonstration, based on our analysis of *rad-50* mutants, that the meiotic program in Caenorhabditis elegans involves both acquisition and loss of a specialized mode of double-strand break repair (DSBR). In premeiotic germ cells, RAD-50 is not required to load strand-exchange protein RAD-51 at sites of spontaneous or ionizing radiation (IR)-induced DSBs. A specialized meiotic DSBR mode is engaged at the onset of meiotic prophase, coincident with assembly of meiotic chromosome axis structures. This meiotic DSBR mode is characterized both by dependence on RAD-50 for rapid accumulation of RAD-51 at DSB sites and by competence for converting DSBs into interhomolog COs. At the mid-pachytene to late pachytene transition, germ cells undergo an abrupt release from the meiotic DSBR mode, characterized by reversion to RAD-50-independent loading of RAD-51 and loss of competence to convert DSBs into interhomolog COs. This transition in DSBR mode is dependent on MAP kinase-triggered prophase progression and coincides temporally with a major remodeling of chromosome architecture. We propose that at least two developmentally programmed switches in DSBR mode, likely conferred by changes in chromosome architecture, operate in the C. elegans germ line to allow formation of meiotic crossovers without jeopardizing genomic integrity. Our data further suggest that meiotic cohesin component REC-8 may play a role in limiting the activity of SPO-11 in generating meiotic DSBs and that RAD-50 may function in counteracting this inhibition.

## Author Summary

Faithful inheritance of chromosomes during sexual reproduction depends on the deliberate formation of double-strand DNA breaks (DSBs) and subsequent repair of a subset of these breaks by a mechanism that leads to crossovers between homologous chromosome pairs. The requirement for crossovers to ensure chromosome segregation poses a challenge for sexually reproducing organisms, as DSBs constitute a danger to genomic integrity in other contexts. This manuscript provides insight into the mechanisms that allow germ cells to generate recombination-based linkages that ensure chromosome inheritance while at the same time protecting the integrity of their genomes. Specifically, we provide a direct demonstration, based on our analysis of *rad-50* mutants, that the meiotic program in C. elegans involves both acquisition and loss of a specialized meiotic mode of double-strand break repair (DSBR). We propose that the ability to revert to a less constrained DSBR environment at a late stage of meiotic prophase serves as a fail-safe mechanism for safeguarding the genome, as it provides an opportunity to repair any remaining DBSs and restore chromosome integrity prior to chromosome segregation.

## Introduction

Faithful inheritance of chromosomes during meiosis relies on crossover (CO) recombination events between the DNA molecules of homologous chromosomes. Interhomolog COs underpin the formation of chiasmata that temporarily link homologs and allow them to orient and segregate toward opposite poles of the meiosis I spindle [1]. This requirement for crossovers to ensure homolog segregation poses a challenge for sexually reproducing organisms, however, as meiotic recombination is initiated by formation of double-strand DNA breaks (DSBs) [2], lesions that constitute a danger to genomic integrity in other contexts. Thus, it is crucial that germ cells possess mechanisms not only for converting a subset of meiotic DSBs into interhomolog COs but also for limiting the number of DSBs formed and for repairing any excess DSBs prior to the meiotic cell divisions.

As interhomolog COs are rare during mitotic cell cycles, the need for specialized features that promote crossing over between homologs during meiosis has long been apparent. Consequently, research in a variety of experimental systems has yielded substantial knowledge regarding components of the machinery and mechanisms involved in promoting meiotic crossing over. However, relatively little attention has been focused on the importance of mechanisms that can constrain the activity of Spo11, the DSB-forming endonuclease [2]. Likewise, the idea that germ cells might possess mechanisms to inactivate features of the meiotic recombination program that serve as impediments to DSB repair (DSBR) has not been widely articulated. Although we had previously proposed that distinct modes of DSBR might operate during different stages of meiotic prophase in C. elegans to ensure restoration of intact chromosomes [3,4], the prior evidence for this assertion was indirect and largely circumstantial.

In the current work, we now provide a direct demonstration that the meiotic program in C. elegans germ cells involves both acquisition and loss of a specialized mode of DSBR during meiotic prophase progression. This conclusion emerged during the course of analyzing DNA damage responses in mutants defective in *rad-50,* which encodes a component of the conserved Mre11/Rad50 complex that has been implicated in numerous aspects of both meiotic recombination programs and the DNA damage response in mitotically dividing cells [5–8]. The spatial organization of the C. elegans germ line was instrumental in this analysis. The fact that germ cells undergoing mitotic proliferation and germ cells entering and progressing through meiotic prophase are arranged in a temporal/spatial gradient along the distal-proximal axis of the gonad enabled simultaneous visualization of responses to DNA damage in germ cells at all stages of meiotic prophase. Further, this organization also enabled us to perform a “reverse time course” analysis in which we assessed outcomes for germ cells that were at progressively earlier stages of meiotic prophase at the time of exposure to an acute break-inducing treatment.

Our analysis shows that germ cells switch abruptly to a specialized meiotic mode of DSBR at the onset of meiotic prophase, visualized cytologically as acquisition of a requirement for RAD-50 to allow rapid accumulation of DNA strand exchange protein RAD-51 at DSB sites. Moreover, we show that germ cells undergo a second developmentally-programmed switch in DSBR mode as they pass through a MAP kinase-dependent transition from the mid-pachytene to late pachytene stage of meiotic prophase [9]. This second switch is characterized by loss of RAD-50 dependence for RAD-51 loading and by loss of competence to convert DSBs into interhomolog COs. Each of these switches coincides temporally with a major remodeling of chromosome architecture [10–12], supporting the view that specialized aspects of the meiotic DSBR program are imposed or enforced by meiosis-specific differentiation of chromosome axis structures. Further, we also provide evidence suggesting that meiosis-specific cohesins may play an inhibitory role that limits the formation of meiotic DSBs. Together these features help to explain how germ cells are able to balance the imperative to generate recombination-based linkages that ensure chromosome inheritance with the imperative to preserve the integrity of their genomes.

## Results

### *C. elegans rad-50* Mutants Are Defective for Chiasma Formation During Meiosis

The *rad-50* gene encodes the C. elegans ortholog of eukaryotic Rad50 and bacterial SbcC. Based on the structure of its orthologs, the N- and C-terminal domains of RAD-50 are predicted to comprise a bipartite ABC-type ATPase domain, with the intervening portion of the protein forming an extended intramolecular α-helical coiled-coil and a zinc-coordinating “hook” dimerization motif located at the end of the coiled-coil distant from the ATPase domain [13–15]; this structural organization is similar to that of the SMC components of the condensin and cohesin complexes [16]. RAD-50 associates with conserved SbcD nuclease-domain protein MRE-11 [17], and complexes containing their orthologs have demonstrated endonuclease, exonuclease, DNA unwinding, and end-tethering activities in vitro [5–8]. The *rad-50(ok197)* allele contains a 1,517 bp deletion that removes the first 1,065 bp of coding sequence plus 124 bp immediately upstream of the predicted initiation codon. As this deletion eliminates the exons encoding the entire N-terminal portion of the ATPase domain (including the Walker A box) and about one-third of the N-terminal segment of the coiled-coil domain (which interacts with MRE-11), *rad-50(ok197)* is predicted to be a null allele.

**Figure 1:**
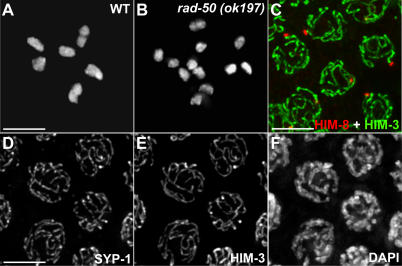
RAD-50 Is Required for Chiasma Formation but Not for Homolog Pairing and Synapsis (A, B) Each panel shows the full karyotype in a single oocyte nucleus at diakinesis, the last stage of meiotic prophase. The wild-type nucleus (A) contains six DAPI-stained bodies, corresponding to the six pairs of homologous chromosomes (bivalents) attached by chiasmata, whereas the *rad-50* mutant nucleus (B) contains 12 separate DAPI-stained chromosomes (univalents), indicating a lack of chismata connecting homolog pairs. (C) Pachytene nuclei from the *rad-50* mutant stained with antibodies detecting X-chromosome pairing center-binding protein HIM-8 and chromosome axis protein HIM-3. A single HIM-8 focus (or two closely spaced HIM-8 foci) is seen in each nucleus, indicating successful homologous pairing. (D–F) Pachytene nuclei from the *rad-50* mutant, showing coimmunolocalization of SC central region protein SYP-1 and meiotic chromosome axis protein HIM-3 at the interface between parallel tracks of DAPI-stained chromatin, indicating successful assembly of the SC. Scale bar = 5 μm.

Homozygous *rad-50* mutant hermaphrodites from heterozygous (*rad-50/+)* mothers are viable but exhibit a set of phenotypes characteristic of mutants defective in meiotic recombination, similar to those previously reported for *C. elegans mre-11* mutants [18]. They produce 98.4% dead embryos (mainly reflecting aneuploidy resulting from autosomal missegregation) and a high incidence of XO males among the surviving progeny (the “Him” phenotype, reflecting X chromosome missegregation) (Table 1). Further, cytological examination of oocyte nuclei at diakinesis (the last stage of meiotic prophase) indicates that this chromosome segregation defect results from a lack of chiasmata connecting homologous chromosomes (Figure 1), presumably reflecting a failure to form interhomolog crossovers.

**Table 1:**
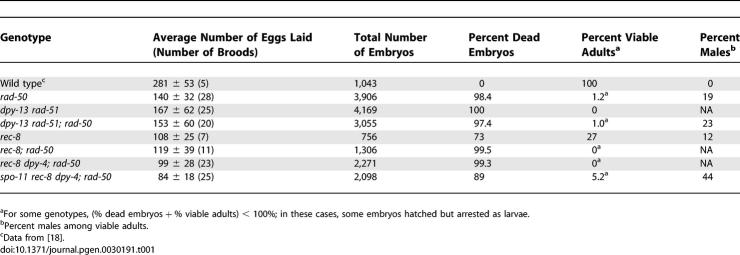
Progeny Viability and Percent Males

DAPI staining, immunofluorescence, and fluorescence in situ hybridization (FISH) experiments revealed apparently normal pairing and synapsis of homologous chromosomes in *rad-50* mutant germ lines. As in wild-type meiosis [11], chromosome axis protein HIM-3 and synaptonemal complex (SC) central region protein SYP-1 colocalized at the interface between parallel-aligned DAPI tracks in pachytene nuclei of *rad-50* mutants, indicating successful assembly of the SC (Figure 1). Further, we detected a single focus or two closely-spaced foci in pachytene nuclei both in FISH experiments assessing pairing at the 5S rDNA locus on chromosome V (Figure S1) and in experiments assessing pairing at the pairing center (PC) domain of the X chromosomes (by immunofluorescence of PC-binding protein HIM-8 [19,20]; Figure 1). The success of homologous pairing and synapsis indicates that the defect in chiasma formation in *rad-50* mutants most likely results from a defect in the process of recombination per se.

### Altered Patterns of Both SPO-11-Dependent Meiotic RAD-51 Foci and Spontaneous (SPO-11-Independent) RAD-51 Foci in *rad-50* Mutant Germ Lines

**Figure 2:**
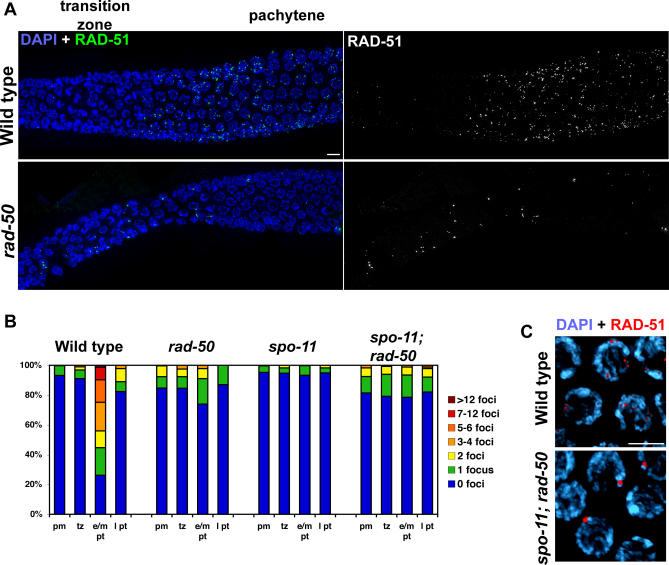
Altered Profile of RAD-51 Foci in *rad-50* Mutant Germ Lines (A) Images show a portion of the germ lines of wild type and *rad-50* mutant hermaphrodites, extending from the premeiotic region in which nuclei have not yet entered meiotic prophase (left) through the region of meiotic prophase entry (transition zone) and into the pachytene region (in which chromosomes are fully paired and synapsed; right). Chromosomes are stained with DAPI and RAD-51 antibody. In the wild-type germ line, RAD-51 foci are very infrequent in premeiotic and transition zone nuclei, but are abundant in pachytene nuclei, with multiple foci in the majority of nuclei. In the *rad-50* mutant germ line, the number of foci in premeiotic and transition zone nuclei is elevated, while the number of foci in pachytene nuclei is severely reduced. Scale bar = 5 μm. (B) Quantitative time course analysis of RAD-51 foci. Stacked bar graph depicting quantitation of RAD-51 foci in premeiotic nuclei (pm), transition zone nuclei (tz), early-mid pachytene nuclei (e/m pt), and late pachytene nuclei (l pt) in gonads of the indicated genotypes. Different colored segments represent the percentage of nuclei scored that had the numbers of RAD-51 foci indicated by the color code at the right of the graph. (C) High magnification view of pachytene nuclei from wild type and *spo-11; rad-50* germ lines. Whereas the RAD-51 foci in the wild-type pachytene nuclei are more numerous, the SPO-11-independent RAD-51 foci in the *spo-11; rad-50* nuclei are larger/brighter. Scale bar = 5 μm.

Immunostaining for DNA strand exchange protein RAD-51 [3,21] revealed abnormalities in *rad-50* mutant germ lines. First, *rad-50* mutants exhibited severely reduced levels of SPO-11-dependent meiotic RAD-51 foci (Figure 2A and 2B). We quantified the number of foci stained with RAD-51 antibody in premeiotic germ cells and in germ cells entering and progressing through meiotic prophase. In wild-type control germ lines, levels of RAD-51 foci rose following entry into meiotic prophase and peaked in the early to mid-pachytene stage. Numbers of RAD-51 foci were greatly reduced in nuclei at the corresponding stages of meiotic prophase in *rad-50* mutants, paralleling previous reports of reduced RAD-51 foci in *mre-11* mutants [21]. This reduction in meiotic prophase RAD-51 foci indicates either that meiotic DSB formation is reduced in *rad-50* mutants or that RAD-50 is required to accumulate RAD-51 at meiotic DSBs.

Second, *rad-50* mutants exhibited elevated levels of SPO-11-independent RAD-51 foci (Figure 2B). Although levels of RAD-51 foci in meiotic prophase nuclei were severely reduced in the *rad-50* mutant, residual levels of RAD-51 foci were higher than in *spo-11* mutant germ lines (which lack meiotic DSBs [3,22]), and numbers of foci in premeiotic nuclei were elevated compared to either wild-type or *spo-11* controls. *spo-11; rad-50* double mutant germ lines exhibited RAD-51 focus profiles similar to those of the *rad-50* single mutant, indicating that most (if not all) of the RAD-51 foci observed in *rad-50* mutants were SPO-11-independent in origin (Figure 2B). Further, the SPO-11-independent foci observed both in the premeiotic and meiotic prophase regions of *rad-50* and *spo-11; rad-50* mutant germ lines appeared larger and brighter than either the SPO-11-dependent foci or premeiotic foci observed in controls (Figure 2C and unpublished data). These bright SPO-11-independent RAD-51 foci likely represent spontaneous DNA breaks and/or single-strand gaps that arose during the course of DNA replication; their presence suggests that RAD-50 is not required to load RAD-51 at lesions incurred in premeiotic nuclei. Further, the SPO-11-independent RAD-51 foci observed in meiotic prophase nuclei likely represent persistence of unrepaired breaks that occurred prior to meiotic prophase entry.

Consistent with the presence of unrepaired DSBs, the *rad-50* mutant exhibited elevated levels of apoptosis in the late pachytene region of the germ line, where the pachytene DNA damage checkpoint operates to cull nuclei with persistent DNA damage [23]. Using Nomarski differential interference contrast (DIC) microscopy to score apoptosis in adult (24–28 h post L4) germ lines [24], we observed an average of 4.5 apoptotic nuclei per gonad arm in *rad-50(ok197)* mutants (*n* = 24 gonad arms), compared with an average of 1.1 apoptotic nuclei per gonad arm in wild-type controls (*n* = 21 gonad arms) (*p* < 0.0001 for Mann-Whitney test).

### Context-Dependent Requirement for RAD-50 for Rapid Accumulation of RAD-51 at Irradiation-Induced DSBs

**Figure 3:**
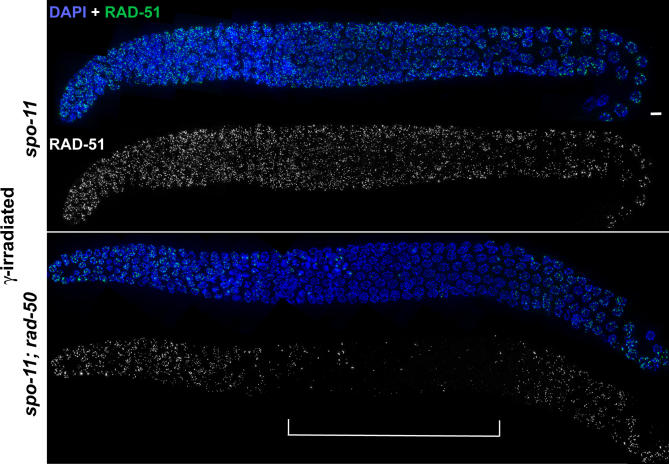
Context-Specific Requirement for Rapid Accumulation of RAD-51 at Sites of IR-Induced Breaks Germ lines from *spo-11(ok79)* and *spo-11(ok79); rad-50* hermaphrodites fixed 1 h after exposure to 1 krad γ-irradiation and stained with DAPI and RAD-51 antibody. RAD-51 foci are detected in germ cell nuclei throughout the *spo-11* gonad, from the proliferating nuclei at the distal tip (left) through nuclei at the diplotene stage of meiotic prophase (right). In the *spo-11; rad-50* mutant, in contrast, RAD51 foci are absent in most nuclei in the central portion of the gonad, indicated by the bracket, from the onset of meiotic prophase through the mid-pachytene/late pachytene transition (see Figure 4). Scale bar = 5 μm.

Experiments aimed at assessing the response of *rad-50* mutants to ionizing radiation (IR) unexpectedly revealed that C. elegans germ cells switch abruptly between RAD-50-independent and RAD-50-dependent modes of accumulation of RAD-51 at DNA break sites as they enter and progress through meiotic prophase. To focus on IR-induced breaks in the absence of endogenous meiotic breaks, we exposed *spo-11* control and *spo-11; rad-50* double mutant worms to 1,000 rad γ-irradiation and dissected and fixed their gonads 1 h post irradiation. Immunostaining revealed abundant RAD-51 foci in nuclei throughout the germ lines of irradiated *spo-11* single mutant controls and irradiated wild-type worms (Figures 3 and S2), indicating that premeiotic germ cells and germ cells at all stages of meiotic prophase are capable of installing RAD-51 onto chromosomes, presumably at break sites. Similar to these controls, the germ lines of *spo-11; rad-50* mutant worms also exhibited abundant IR-induced RAD-51 foci both in the premeiotic region and in the region containing nuclei from late pachytene through diakinesis stages of meiotic prophase (Figure 3). In contrast to controls, however, irradiated *spo-11; rad-50* double mutant germ lines contained a region extending from the transition zone (where nuclei enter meiotic prophase) through mid-pachytene in which most nuclei were devoid of RAD-51 foci; within this region, germ lines appeared similar to the corresponding regions of unirradiated *spo-11; rad-50* controls (i.e., with a subset of nuclei containing 1–2 bright spontaneous RAD-51 foci).

**Figure 4:**
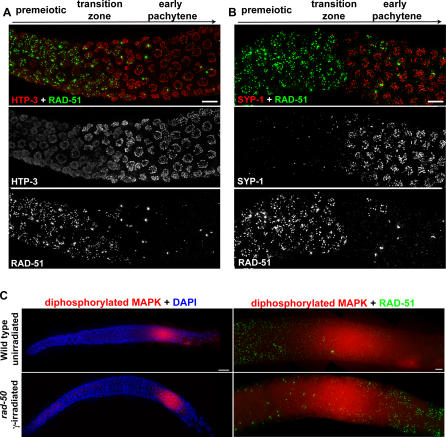
RAD-50 Dependence for Loading of RAD-51 at IR-Induced Breaks Extends from Meiotic Prophase Onset to the Mid-Pachytene/Late Pachytene Transition (A, B) Images show a portion of the germ lines of *rad-50* mutant hermaphrodites fixed 1 h after exposure to 1 krad γ-irradiation, centered on the transition zone and extending from prior to the onset of meiotic prophase (premeiotic, left) through early pachytene (right). (A) HTP-3 staining is diffusely associated with chromatin in premeiotic nuclei, then becomes concentrated on chromosome axis structures at the beginning of the transition zone, first appearing as discontinuous stretches and then as extended continuous linear structures. IR-induced RAD-51 foci are abundant in nuclei with diffuse HTP-3 staining, whereas nuclei with concentrated meiotic HTP-3 signals have very few RAD-51 foci. Scale bar = 5 μm (B) SYP-1 immunostaining is either absent or detected as a single bright focus in premeiotic nuclei, and is extensively localized on chromosomes beginning in the transition zone. IR-induced RAD-51 foci are abundant in nuclei lacking SYP-1, whereas nuclei with abundant SYP-1 have very few RAD-51 foci. Scale bar = 5 μm. (C) An unirradiated wild-type gonad and an irradiated *rad-50* gonad stained with DAPI, α-RAD-51, and a monoclonal antibody detecting the activated, diphosphorylated form of MAP kinase (MAPK). Left, zoomed out view of the gonads (distal tip through diplotene), showing the position of the peak of MAP kinase activation that occurs at the mid-pachytene to late pachytene transition. Right, zoomed in view of the region surrounding this peak of activated MAP kinase, showing the location of this peak relative to RAD-51 foci. In the unirradiated wild-type germ line, RAD-51 foci peak in mid-pachytene and diminish in numbers prior to the peak of activated MAPK. In the irradiated *rad-50* germ line, background levels of bright SPO-11-independent spontaneous RAD-51 foci are seen in a subset of nuclei throughout the gonad, whereas IR-induced RAD-51 foci abruptly rise in abundance in late pachytene nuclei, after the peak of activated MAPK. Left; Scale bar = 20 μm. Right; Scale bar = 5 μm.

An identical “dark zone” lacking IR-induced RAD-51 foci was also seen in the germ lines of *rad-50* and *mre-11* single mutant worms exposed to 1 krad γ-irradiation (Figures 4 and S2); similar observations for the *mre-11* mutant were also made in an independent study (A. Penkner and J. Loidl, personal communication.). Further, the contrast between the “dark zone” and regions with abundant IR-induced RAD-51 foci was even more pronounced following exposure of *rad-50* mutants to a 5 krad dose (Figure S3). Interestingly, the region of the germ line where IR-induced foci were lacking in irradiated *spo-11; rad-50* and *rad-50* worms corresponds to the region where meiotic RAD-51 foci are most abundant in unirradiated wild-type germ lines (Figures 2A and 4; [3]).

We will operationally use the term “RAD-51 loading” to refer to the rapid formation of RAD-51 foci following break-inducing treatment. Although both association and dissociation of RAD-51 subunits will contribute to the immunofluorescence signals observed, evidence presented below supports the interpretation that slow/delayed formation of RAD-51 foci, rather than accelerated turnover of RAD-51-containing complexes, is responsible for the lack of IR-induced RAD-51 foci in germ cells within the “dark zone” of *rad-50* mutant gonads.

### Acquisition of the RAD-50-Dependent Mode of RAD-51 Loading Coincides with Meiotic Prophase Onset

Simultaneous immunolocalization of RAD-51 and meiotic chromosome structural proteins in the distal germ lines of IR-treated *rad-50* worms demonstrated that a switch from RAD-50 independence to RAD-50 dependence for RAD-51 loading occurs at the onset of meiotic prophase. The earliest marker of meiotic prophase is concentration of HTP-3 protein onto nascent chromosome axis structures [19], first detected as discontinuous stretches and then as continuous bright lines. Whereas IR-induced RAD-51 foci were abundant in premeiotic nuclei (which exhibited diffuse HTP-3 staining on chromatin), there was an abrupt transition to a RAD-51-negative state in nuclei that had begun to acquire meiotic organization of HTP-3 structures (Figure 4A). Similar results were obtained in experiments imaging RAD-51 together with SYP-1, which assembles onto chromosomes following initial assembly of chromosome axes [11]. IR-induced RAD-51 foci were abundant in premeiotic nuclei lacking SYP-1 immunofluorescence (IF) signals, but were lacking in nuclei in which SYP-1 was extensively associated with chromosomes (Figure 4B). Together these results indicate that entry into meiotic prophase is accompanied by imposition of a requirement for RAD-50 to allow rapid accumulation of RAD-51 at sites of DNA breaks.

### Reversion to RAD-50 Independence for RAD-51 Loading at the Mid-Pachytene to Late Pachytene Transition

A second transition, in which nuclei revert to RAD-50-independence for loading of RAD-51, is observed as nuclei progress from mid-pachytene to late pachytene regions of the germ line (Figures 3 and 4C). Previous work showed that progression from mid to late pachytene requires a MAP kinase-dependent signaling event, visualized cytologically as a transient rise in the activated, diphosphorylated form of MAP kinase [9,25]. In wild-type germ lines, this peak in activated MAP kinase is found in a position just proximal to the zone in which SPO-11-dependent meiotic RAD-51 foci gradually decline in numbers, presumably reflecting progression of meiotic recombination (Figure 4C). The peak of activated MAP kinase was found in a similar position in the germ lines of irradiated *rad-50* mutants (Figure 4C), indicating that activation of MAP kinase in this context does not require RAD-50. Moreover, the position of the peak is just distal to the abrupt transition to high levels of IR-induced RAD-51 foci. Further, the germ lines of irradiated *mpk-1 (ga111ts); rad-50* double mutants, in which meiotic prophase progression was arrested at the mid-pachytene stage, did not exhibit a rise in levels of IR-induced RAD-51 foci in the corresponding region (Figure S4). Together these data indicate that reversion to a RAD-50-independent mode of RAD-51 loading requires MAP kinase-dependent developmental progression from the mid-pachytene to late pachytene stage. We will use the term “constrained region” to refer to the region of the germ line, extending from the onset of meiotic prophase to the mid-pachytene to late pachytene transition, in which germ cells exhibit the requirement for RAD-50 to achieve rapid accumulation of RAD-51.

### Time Course Analysis of IR-Induced RAD-51 Foci

**Figure 5:**
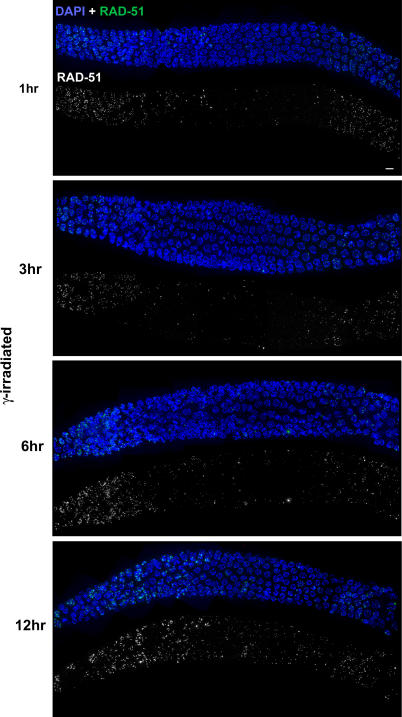
Time Course Analysis of IR-Induced RAD-51 Foci in the Constrained Region of *spo-11; rad-50* Germ Lines Images show portions of *spo-11; rad-50* germ lines, centered on the constrained region and also including a subset of premeiotic nuclei (left) and late pachytene nuclei (right). Germ lines were fixed at the indicated times following exposure to 1 krad γ-irradiation and stained with DAPI and RAD-51 antibody. An obvious RAD-51-dark region in the central segment of the germ line is readily discernable at the 1, 3 and 6 h time points, but not at the 12 h time point. Scale bar = 5 μm.

Examination of *spo-11; rad-50* germ lines that were fixed at additional time points (3, 6 and 12 h) after irradiation supports the interpretation that the dearth of IR-induced RAD-51 foci observed in the constrained region of *spo-11; rad-50* germ lines (and *rad-50* or *mre-11* germ lines) is indicative of a prolonged delay in and/or severely reduced rate of accumulation of RAD-51 at break sites. At both the 3 h and 6 h post-irradiation time points, we continued to observe a robust RAD-51-dark zone in the central portion of *spo-11; rad-50* mutant germ lines, indicating that IR-induced RAD-51 focus formation remained strongly inhibited in germ cells within this region (Figure 5). By 12 h post irradiation, however, a clear RAD-51-dark zone was no longer visible. At this 12 h time point, many transition zone nuclei and some early pachytene nuclei contained multiple bright RAD-51 signals (Figure S5); these bright signals reflect persistence of preassembled RAD-51 foci in nuclei that had been in the premeiotic region at the time of irradiation ([26]; S. Mlynarczyk-Evans and A. Villeneuve, unpublished data). Further, we also observed some smaller/less intense RAD-51 foci in the majority of nuclei within the mid-pachytene region of these germ lines (Figures 5, S5, and S6B); this eventual rise in RAD-51 foci in the mid-pachytene region cannot be explained by movement and developmental progression of nuclei with pre-installed foci, as most nuclei within the mid-pachytene region at the 12 h time point had entered meiotic prophase prior to the time of irradiation ([26]; S. Mlynarczyk-Evans and A. Villeneuve, unpublished data). These observations strongly suggest that installation of RAD-51 at DSB sites in nuclei within the constrained region of *rad-50* mutant germ lines is severely delayed and/or occurs at a substantially reduced rate. Moreover, they argue against the alternative interpretation that failure to detect IR-induced RAD-51 foci in the constrained region is consequence of accelerated turnover of RAD-51-containing complexes.

### Loss of Competence to Convert IR-Induced DSBs into Interhomolog Crossovers Coincides Temporally with the Mid-Pachytene to Late Pachytene Transition

We previously showed that IR-induced DSBs can bypass the requirement for SPO-11 in initiating meiotic recombination, leading to formation of crossovers and functional chiasmata in *spo-11* mutants [22,27]. In these prior experiments, the ability of IR-induced breaks to form chiasmata was assessed by examining diakinesis-stage oocytes 18 hrs following irradiation; based on a recent temporal analysis of meiotic prophase progression [26], we infer that the oocytes scored had been in the mid-pachytene stage at the time of irradiation.

To assess whether the transition from mid to late pachytene affects the ability to convert breaks into chiasmata, we performed a “reverse time course analysis” in which we assessed the efficacy for chiasma formation of breaks induced by IR at several time points spanning this transition, ranging from 18 to 12 h prior to fixation for scoring at late diakinesis (Table 2). Chiasmata were efficiently generated by IR delivered at the mid-pachytene time point (18 h); an average of 6.5 DAPI-stained bodies were detected, indicating that all six chromosome pairs were linked by chiasmata in most nuclei. In contrast, IR breaks introduced at the late pachytene time point (12 h) were almost completely ineffective at triggering chiasma formation; an average of 11.4 DAPI-stained bodies were detected, similar to the 11.5 average seen in the unirradiated control. Further, a bimodal distribution in the numbers of DAPI-stained bodies per nucleus was evident at all time points, suggesting that germ cells undergo an abrupt transition between chiasma-competent and chiasma-incompetent states at the mid-pachytene to late pachytene transition.

**Table 2:**
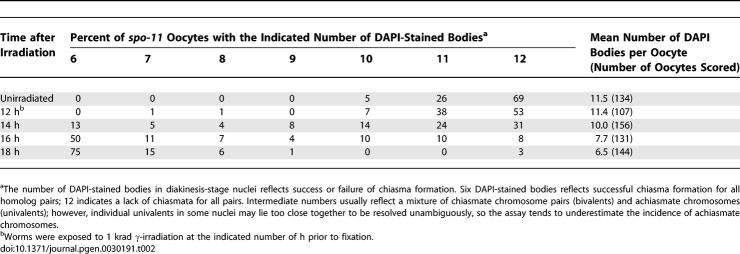
Timing of γ-Irradiation Affects Efficacy of Chiasma Formation in *spo-11* Mutant Germ Cells

### RAD-50 Dependence for RAD-51 Loading Is Partially Abrogated in *htp-1* and *him-3* Mutants

**Figure 6:**
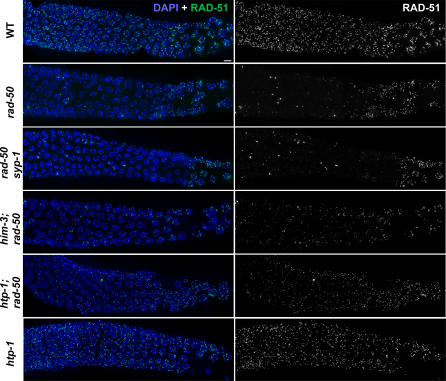
RAD-50 Dependence for RAD-51 Loading Is Partially Abrogated in *him-3* and *htp-1* Mutants Images show portions of germ lines extending from mid pachytene (left) through late pachytene/early diplotene (right) from hermaphrodites of the indicated genotypes that were exposed to 1 krad γ-irradiation 1 h prior to fixation. Both the *rad-50* single mutant and the *rad-50 syp-1* double mutant germ lines exhibit a “dark” region in which most nuclei lack RAD-51 foci, reflecting engagement of the RAD-50-dependent mode of RAD-51 loading that operates from meiotic prophase onset until the mid-pachytene to late pachytene transition. In the *rad-50 syp-1* double mutant, the transition to nuclei with abundant IR-induced RAD-51 foci is observed at a more proximal position than in the *rad-50* single mutant, at the very end of the pachytene stage, following exit from the persistent clustered chromosome configuration that is characteristic of mutants lacking SC central region components. In the *him-3; rad-50* and *htp-1; rad-50* double mutant germ lines shown, IR-induced RAD-51 foci are detected in nuclei throughout the mid-pachytene region, indicating that the requirement for RAD-50 is partially alleviated; however these IR-induced foci are less intense than those seen in the comparable regions of either the wild-type or *htp-1* single mutant control germ lines. Scale bar = 5 μm.

As the onset of the requirement for RAD-50 to load RAD-51 at IR-induced breaks coincides with assembly of meiosis-specific chromosome structures, we tested whether loss of components of these structures might eliminate or mitigate this requirement. First, we found that loss of SC central region proteins does not alleviate this requirement. The overall RAD-51 IF pattern in both unirradiated and irradiated *rad-50 syp-1* germ lines appeared very similar to *rad-50* single mutant counterparts (Figure 6; unpublished data). The one notable difference was that the size of the zone of late pachytene nuclei exhibiting RAD-50-independent RAD-51 loading was reduced in the *rad-50 syp-1* double mutant; the transition to the RAD-50-independent mode correlated with exit from the persistent state of chromosome clustering characteristic of *syp-1* mutants [11].

In contrast, we found that RAD-50 dependence within the constrained region was partially abrogated in both *him-3; rad-50* and *htp-1; rad-50* double mutants (Figures 6 and S3). HIM-3 and HTP-1 are two of four C. elegans paralogs of the meiosis-enriched HORMA domain protein family that also includes Saccharomyces cerevisiae Hop1 and *Arabidpopsis* Asy1 and Asy2 [4,10,28–31]. Both proteins localize to meiotic chromosome axes prior to SC assembly and persist in association with chromosome axes after SC disassembly, and both have been hypothesized to play roles in inhibiting use of the sister chromatid as a DSBR partner during meiotic prophase ([4,10,12,30]; E. Martinez-Perez, A. Dernburg and A. Villeneuve, unpublished data). In the absence of irradiation, RAD-51 IF patterns in *him-3; rad-50* and *htp-1; rad-50* germ lines appeared similar to that of the *rad-50* single mutant (unpublished data). Following IR treatment, however, we observed RAD-51 IF patterns in *htp-1; rad-50* and *him-3; rad-50* mutant germ lines that were intermediate in appearance between those of irradiated *htp-1* or *him-3* single mutants and those observed in irradiated *rad-50* single mutants. Whereas over 120 *rad-50* single mutant gonads examined invariably exhibited a robust inhibition of IR-induced RAD-51 foci throughout the constrained region (extending from the zone of meiotic entry through the mid-to-late pachytene transition), IR-induced RAD-51 foci were frequently detected in nuclei within the constrained region in *htp-1; rad-50* mutant germ lines. In 35% of *htp-1; rad-50* gonads examined following a 1 krad IR treatment (*n* = 26; *p* < 0.0001) and in 97% of *htp-1; rad-50* gonads examined following a 5 krad IR treatment (*n* = 32; *p* < 0.0001), we detected IR-induced RAD-51 foci in nuclei throughout most or all of the mid-pachytene region (Figures 6 and S3). In contrast to *htp-1* single mutants, however, all *htp-1; rad-50* gonads contained at least a small domain of transition zone and/or early pachytene nuclei in which IR-induced RAD-51 foci were strongly inhibited (Figure S3). Similarly, 36% of *him-3; rad-50* gonads exposed to 1 krad IR treatment clearly exhibited IR-induced RAD-51 foci within the constrained region (*n* = 50; *p* < 0.0001). However, the altered pattern observed in *him-3; rad-50* gonads differed from that seen in *htp-1; rad-50* gonads in that when IR-induced RAD-51 foci were detected within the constrained region, they were present in nuclei throughout the entire region. In both cases, the IR-induced foci within the constrained region appeared smaller and/or less intense than those in either the premeiotic region or in late pachytene, suggesting that RAD-51 loading was slower and/or occurred over more limited stretches. The fact that RAD-50 dependence is only partially abrogated in *him-3* or *htp-1* mutant backgrounds may be a consequence of partial redundancy among the meiosis-enrich HORMA domain proteins, as all four C. elegans paralogs show similar localization to meiotic chromosomes ([4,10,19,30]; E. Martinez-Perez, A. Dernburg and A. Villeneuve, unpublished data).

Partial alleviation of the requirement for RAD-50 within the constrained region in these double mutants strongly suggests that proteins associated with meiosis-specific chromosome axis structure play a role in imposing this requirement. Further, the pattern of IR-induced foci observed in the *htp-1; rad-50* double mutants suggests that germ cells lacking HTP-1 can at least partially engage the meiotic DSBR program at the onset of meiotic prophase but then undergo a premature release from the constraints that make rapid accumulation of RAD-51 dependent on RAD-50. Interestingly, this finding and interpretation parallels the previously proposed hypothesis that HTP-1 is required to prevent premature release from meiotic prophase constraints that inhibit use of sister chromatids as meiotic recombination partners [4].

### RAD-50 and Meiotic DSB Formation

In light of our finding that RAD-50 is required within the constrained region for normal accumulation of RAD-51 at DSB sites, it was necessary to revisit the question of whether RAD-50 is also required for meiotic DSB formation. Collectively, the data reported in this section suggest that although RAD-50 likely plays a role in promoting normal levels of meiotic DSB formation, it is not strictly required for SPO-11 to be active in generating DSBs.

**Figure 7:**
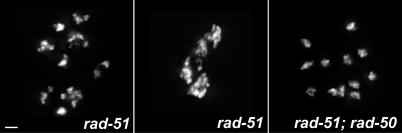
Abnormal Chromosome Morphology Associated with *rad-51* Mutants Is Suppressed in *rad-51; rad-50* Double Mutants Each panel shows the full karyotype in a single diakinesis-stage oocyte. Diakinesis oocytes in the *rad-51* mutant typically have abnormal-appearing, poorly condensed chromosomes (left) and/or aggregated masses of chromosomes (middle), whereas diakinesis oocytes in the *rad-51; rad-50* double mutant exhibit well-formed, non-aggregated univalent chromosomes (right). Scale bar = 2 μm.

Several lines of evidence support the conclusion that SPO-11dependent DSB formation is reduced in *rad-50* mutants. First, we did not observe a rise in abundance of RAD-51 foci in late pachytene or diplotene nuclei in unirradiated *rad-50*, *rad-50 syp-1*, *him-3; rad-50* or *htp-1; rad-50* germ lines (Figure 2; unpublished data); such a rise might have been expected following the transition to late pachytene if chromosomes had experienced SPO-11-generated breaks during earlier prophase. Second, analysis of *rad-51; rad-50* double mutants also suggests that meiotic DSBs are reduced in number in *rad-50* mutants. The *rad-50* mutation suppresses the embryonic lethality of a *rad-51* mutant [21] (Table 1): whereas *rad-51* mutant hermaphrodites produced no viable embryos (*n* = 4,169), *rad-51; rad-50* hermaphrodites produced 1% viable progeny (*n* = 3,055), similar to *rad-50* single mutants. Most progeny lethality associated with the *rad-50* mutation can be explained as a consequence of aneuploidy resulting from meiotic missegregation, whereas the complete lethality of the *rad-51* progeny is thought to be a combined consequence of both missegregation and failed and/or defective repair of meiotic DSBs [21,32]; thus suppression of *rad-51* progeny lethality by the *rad-50* mutation suggests that meiotic DSBs are diminished in number by the *rad-50* mutation. Similarly, the *rad-50* mutation also suppresses cytological correlates of DSB formation visible in diakinesis-stage oocytes of *rad-51* mutants (Figure 7). Aggregated, poorly condensed chromatin masses are frequently observed in diakinesis-stage oocytes in *rad-51* mutant and in *rad-51(RNAi)* worms [21,33,34]; this pathology is apparently a consequence of defective repair of meiotic DSBs, as it is suppressed by a *spo-11* mutation. The *rad-51* aggregated chromatin phenotype is similarly suppressed by the *rad-50* mutation, as distinct univalents are seen in *rad-51; rad-50* diakinesis nuclei.

Although suppression of the *rad-51* defects by the *rad-50* mutation is most simply explained by a reduction in meiotic DSBs, suppression is also consistent with the *rad-50* mutation allowing use of alternative repair pathways and/or preventing entry of DSBs into a pathological dead-end pathway. This alternative explanation seems less likely to account for the above-mentioned lack of a late-prophase rise in RAD-51 foci in most unirradiated *rad-50* single and double mutants, however, given that RAD-51 protein is present and can eventually be loaded at IR-induced DSBs incurred during early meiotic prophase (albeit with a substantial delay). Thus, taken together these observations suggest that SPO-11dependent DSB formation may be abrogated in *rad-50* mutants.

**Figure 8:**
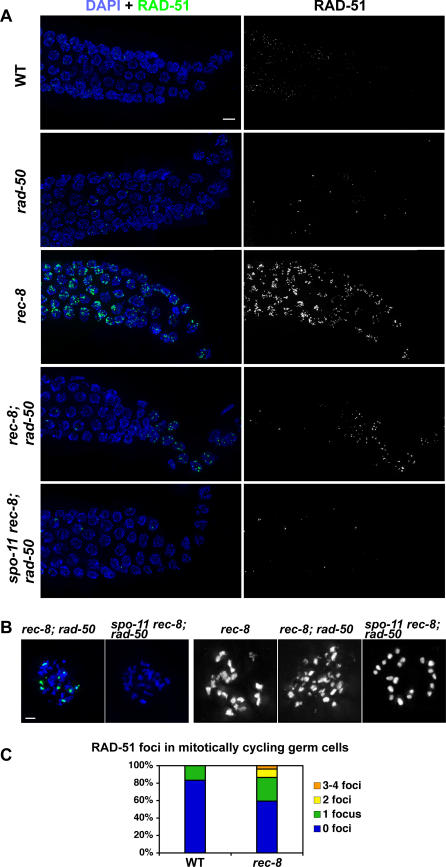
DSB Formation and RAD-51 Loading in the *rec-8* Mutant Background (A) Images show portions of the germ lines extending from mid-late pachytene through diplotene from worms of the indicated genotypes (these worms were NOT exposed to IR). Persistent high levels of meiotic RAD-51 foci are observed throughout this region in the *rec-8* single mutant [full genotype: *rec-8(ok978) dpy-4(e1166)*]. In the *rec-8; rad-50* double mutant [full genotype: *rec-8(ok978) dpy-4(e1166); rad-50(ok197)*], a region in which most nuclei lack RAD-51 foci (left) is followed by a region in which most nuclei have multiple RAD-51 foci. The transition between these states in the unirradiated *rec-8; rad-50* gonad occurs at the very end of the pachytene region. This late pachytene rise in RAD-51 foci is absent in the *spo-11 rec-8; rad-50* triple mutant [full genotype: *spo-11(me44) rec-8(ok978) dpy-4(e1166); rad-50(ok197)*], implying that these late foci are SPO-11-dependent. (B) Each panel shows the full karyotype in a single oocyte nucleus of the indicated genotype, either at the late diplotene/early diakinesis stage (left two panels, stained with DAPI [blue] and RAD-51 antibody [green]) or at late diakinesis (stained with DAPI in white; images shown are from the most mature oocyte in the gonad arm, in the −1 position immediately adjacent to the spermatheca). Multiple RAD-51 foci are detected in the late diplotene/early diakinesis nucleus from the *rec-8; rad-50* worm, whereas the *spo-11 rec-8; rad-50* nucleus at the same stage lacks RAD-51 foci. Similarly, multiple chromosome fragments are evident in the late diakinesis nucleus from the *rec-8; rad-50* worm, whereas such fragments are not detected in the *spo-11 rec-8; rad-50* late diakinesis nucleus (in which chromosomes are present largely as prematurely resolving pairs of sister chromatids). Scale bar = 2 μm. (C) Elevated levels of premeiotic RAD-51 foci in *rec-8* mutant germ lines. RAD-51 foci were quantified for nuclei within the first 15 rows of germ cells, beginning at the distal tip of the gonad; three different germ lines were scored for each genotype (185 total nuclei for wild type, 177 total nuclei for *rec-8).* Stacked bar graph depicts the percentages of nuclei scored that had the indicated numbers of RAD-51 foci; the difference between *rec-8* and wild type germ lines is extremely significant (*p* < 0.0001).

Despite this evidence supporting a role for RAD-50 in promoting meiotic DSB formation, however, analysis of *rec-8(ok978); rad-50* double mutant germ lines provided compelling evidence that SPO-11-dependent meiotic DSBs can nevertheless be formed in the absence of functional RAD-50. *rec-8* encodes a meiosis-enriched α-kleisin subunit of cohesin [16,35], and the *rec-8(ok978)* mutation carries a deletion that is predicted to severely reduce or eliminate *rec-8* function. Meiotic prophase nuclei in the germ lines of unirradiated *rec-8(ok978)* single mutant worms exhibited highly elevated levels of RAD-51 foci that persisted through late pachytene, diplotene and early diakinesis stages (Figure 8A), as seen previously for *rec-8 RNAi* and *rec-8(cosuppression)* worms [21].

Several observations clearly indicate that SPO-11-dependent meiotic DSBs are formed in *rec-8; rad-50* double mutant germ cells. First, whereas RAD-51 immunostaining in *rec-8; rad-50* germ lines appeared similar to that in *rad-50* and *spo-11; rad-50* throughout most of meiotic prophase (i.e., with a subset of nuclei exhibiting one or a few bright foci and most nuclei lacking foci), there was a sharp rise in abundance of RAD-51 foci at late pachytene in *rec-8; rad-50* germ lines, and foci persisted at high levels through diplotene and early diakinesis stages (Figure 8A and 8B). This late pachytene rise is SPO-11 dependent, as it does not occur in *spo-11 rec-8; rad-50* triple mutants. Second, diakinesis nuclei in *rec-8; rad-50* mutants exhibited severely fragmented chromosomes and highly abnormal chromosome morphology (Figure 8B). This fragmentation reflects the presence of SPO-11-dependent DSBs, as it is abolished in *spo-11 rec-8; rad-50* triple mutants. Third, whereas *rec-8; rad-50* mutant hermaphrodites do not produce any viable offspring, *spo-11 rec-8; rad-50* hermaphrodites do produce significant numbers of viable offspring (Table 1). Together these findings demonstrate that formation of SPO-11-dependent DSBs does not strictly require RAD-50, at least in the context of the *rec-8* mutant background.

These findings were corroborated by analysis of a *rec-8; mre-11* double mutant, which also exhibited both a sharp rise in and persistence of RAD-51 foci in late prophase and fragmented chromosomes at diakinesis (unpublished data). Further, an independent study similarly found evidence that MRE-11 is dispensable for DSB formation in the context of compromised cohesin (Y. Mamnun, A. Baudrimont, V. Jantsch and J. Loidl, personal communication).

### Evidence for a Premeiotic Function for REC-8

REC-8 protein is detected cytologically in nuclei throughout the distal premeiotic region of the germ line [35,36], although the significance of this premeiotic localization has not been understood. In the course of this analysis, we observed that in addition to exhibiting high levels of meiotic RAD-51 foci, *rec-8* single mutants also exhibited significantly elevated levels of RAD-51 foci in premeiotic germ cells (Figure 8C). This elevation was observed within the distal-most 15 rows of germ cells, most of which are actively undergoing mitotic cell cycles [37,38]. This finding suggests that REC-8 may function in germ cells even prior to the transition to the meiotic mode of DNA replication [26].

## Discussion

### Multiple Roles for RAD-50, before, during, and after Meiotic Recombination

Our analysis has identified roles for C. elegans RAD-50 in multiple events required for genome stability and chromosome inheritance, during germ cell proliferation, within the context of meiotic recombination, and following exit from the meiotic DSB repair mode in late prophase (see below). First, a requirement for RAD-50 is evident in the distal (premeiotic) region of the germ line, where germ cells are undergoing mitotic proliferation, as *rad-50* mutants exhibit an elevated incidence of RAD-51 foci in this region. Further, these *spo-11*-independent RAD-51 foci are brighter than meiotic RAD-51 foci and persist as germ cells enter and progress through the meiotic program. These findings suggest that RAD-50 plays a role in repairing and/or preventing accumulation of DSBs and/or single-stranded DNA during mitotic proliferation, a role that may reflect the previously-demonstrated requirement for C. elegans MRE-11 to recruit DNA damage response proteins ATR, RPA and BARD1 (but *not* RAD-51) to sites of IR-induced DSBs in proliferating germ cells [39,40].

Second, RAD-50 appears to play several distinct roles in promoting the formation of interhomolog crossovers during the meiotic recombination program. During the “constrained” portion of meiotic prophase, RAD-50 is required for rapid accumulation of RAD-51 at sites of DSBs. This requirement likely reflects a role for the MRE-11/RAD-50 complex in promoting DSB resection in this context. Unresected meiotic DSBs accumulate in *rad50s* and *mre11s* (*s*eparation of function) mutants in S. cerevisiae [41,42], and because Spo11 is found covalently attached to the 5′ overhang at DSBs in these mutants [43], it has been proposed that the requirement for Mre11/Rad50 in DSB resection reflects a requirement for removing Spo11p from DNA ends. A role for Mre11/Rad50 in Spo11 removal was further demonstrated by the recent identification of covalently-linked Spo11-oligonucleotide complexes in wild-type meiosis that are both dependent on Mre11/Rad50 and structurally consistent with expectations for products of its demonstrated endonuclease activity [44]; however, these studies left open the question of whether Mre11/Rad50 might also be required to process recombination intermediates after Spo11 removal. Our demonstration that RAD-50 is required for rapid formation of RAD-51 foci at IR-induced breaks during the period when the meiotic mode of DSBR is engaged strongly supports a role for RAD-50 in DSBR during meiosis beyond removal of SPO-11 from DSB ends, likely in promoting continued end resection.

Further, whereas our analysis of *rec-8; rad-50* mutants clearly demonstrates that RAD-50 is not strictly required for SPO-11-dependent meiotic DSB-forming activity, multiple other data suggest that RAD-50 likely does normally play a role in promoting meiotic DSB formation. Rescue of *rad-51*-associated diakinesis chromosome morphology and progeny lethality in *rad-51; rad-50* double mutants as well as the absence of a late prophase rise in RAD-51 foci and the intact appearance of diakinesis univalents in unirradiated *him-3; rad-50* and *rad-50 syp-1* double mutants are most readily explained as reflections of a substantial reduction in DSB formation in the absence of RAD-50, and consequently as evidence that RAD-50 does play a role in promoting meiotic DSB formation in C. elegans. A requirement for Mre11/Rad50 at the DSB step is clearly not a universal feature of the meiotic program, however. Although such a requirement is well-documented in S. cerevisiae [2], no such requirement exists in S. pombe (where persistent meiotic DSBs in *rad50Δ* mutants are detected by gel assays [45]), in Arabidopsis (where loss of Mre11 results in Spo11-dependent chromosome fragmentation [46]), or in Drosophila (where persistent γHis2Av foci are detected in a *rad50* mutant [47]) . Moreover, even in *S. cerevisiae,* Mre11 and Rad50 are present in a protein complex that is distinct from the complexes that contain Spo11 or several non-conserved proteins required for DSB formation [48–50]. Consequently, the reason for the requirement for Mre11/Rad50 in DSB induction has remained largely mysterious.

Our finding that an apparent requirement for C. elegans RAD-50 in meiotic DSB formation is mitigated in a *rec-8* mutant background provides new framework for thinking about this issue. Our observations can be reconciled in the context of a model proposing that REC-8 and RAD-50 promote counterbalancing influences on chromosome structure that create an environment in which regulated DSB formation can occur. Very high levels of RAD-51 foci are observed in the *rec-8* mutant immediately after entry into meiotic prophase ([21]; unpublished data), raising the possibility that in addition to promoting normal/timely repair of meiotic DSBs, REC-8 may also play an inhibitory role that limits the activity of SPO-11 in DSB formation. In one version of this model, RAD-50 would play a role in counteracting the inhibitory effects of REC-8, perhaps through local action at DSB sites; in the absence of the REC-8 inhibitory effect, the requirement for RAD-50 would be (at least partially) alleviated. An implicit assumption of this scenario is that the SPO-11-dependent DSBs detected as RAD-51 foci and chromosome fragments in late prophase in *rec-8; rad-50* germ lines were formed during early prophase (but were not detected because the meiotic requirement for RAD-50 to load RAD-51 is still functional in the *rec-8* mutant background [unpublished data]). An alternative possibility is that the late foci and breaks in the *rec-8; rad-50* mutant might instead reflect abnormally late activity of SPO-11 in the double mutant. This alternative scenario would also imply a role for REC-8 in inhibiting SPO-11-dependent DSB formation, in this case in preventing DSB formation from occurring during late prophase, within a context in which RAD-50 is no longer required to promote SPO-11 activity. In light of the former model, we are intrigued by the possibility that the requirement for Mre11/Rad50 for meiotic DSB formation in budding yeast might similarly reflect a role in counteracting an inhibitory effect of Rec8. Although this possibility has not been investigated, it is interesting to note that chromatin IP experiments have revealed a negative correlation between cohesin binding sites and meiotic DSB sites [51,52].

Our data provide evidence for one additional role for RAD-50 in C. elegans germ cells as they complete prophase and restructure their chromosomes in preparation for the meiotic divisions. Specifically, the fragmented late diakinesis chromosomes and complete progeny lethality observed in *rec-8; rad-50* double mutants indicate that RAD-50 is also required to restore chromosome integrity even after germ cells have undergone a switch to a “post-meiotic” DNA repair mode at the mid-pachytene to late pachytene transition, at least in the context of high levels of breaks and/or altered relationships between sister chromatids.

### Imposition of and Release from a Specialized Meiotic Mode of DSBR Coupled to Changes in Chromosome Architecture

It has long been appreciated that the dependence of meiotic chromosome segregation on interhomolog crossovers requires both (1) an ability to generate meiotic DSBs to serve as initiating events and (2) acquisition of a specialized mode of DSBR that promotes conversion of a subset of DSBs into interhomolog crossovers. Meiotic specializations include mechanisms for inhibiting sister-directed repair as well as recruitment of meiosis-specific recombination proteins that promote the crossover outcome. We had previously proposed that meiotic cells might also possess an “exit strategy” involving a late prophase release from the constraints imposed by the meiotic mode of DSBR and reversion to a default DSBR mode as a means to ensure restoration of intact chromosomes prior to the meiotic divisions [3,4]. A similar idea was suggested by Mahadevaiah et al. [53] to explain the disappearance of DSB-associated markers in chromosome regions undergoing heterosynapsis in mouse spermatocytes. Until recently, however, the evidence for such a late prophase switch in DSBR mode was largely circumstantial and indirect. The analysis of IR-induced RAD-51 focus formation in *rad-50* mutants reported here has provided a means to visualize directly a programmed shift in the requirements for DSBR that is imposed upon meiotic prophase entry and released in late prophase. Specifically, we have demonstrated that a requirement for RAD-50/MRE-11 to achieve rapid accumulation of RAD-51 at DSBs is acquired at the onset of meiotic prophase in C. elegans and is lost following progression of germ cells through a MAP kinase-dependent transition from the mid-pachytene to late pachytene stage.

Our data suggest that the “meiotic DSBR mode” (characterized by RAD-50 dependence for RAD-51 loading) is imposed, at least in part, by meiosis-specific differentiation of chromosome axis structures. The switch to RAD-50 dependent RAD-51 loading is engaged at the onset of prophase and coincides temporally with assembly of meiotic chromosome axes. Further, this dependence is partially alleviated in mutants lacking either HIM-3 or HTP-1, which are major components of meiotic chromosome axes ([10]; E. Martinez-Perez, A. Dernburg and A. Villeneuve, unpublished data). The fact that RAD-50 dependence is partially abrogated in *htp-1* and *him-3* mutants argues against a trivial explanation for RAD-50 dependence, i.e., absence of expression of a compensating nuclease during the window of RAD-50 dependence. In contrast, SC central region assembly is not required to confer RAD-50 dependence, as the requirement persists in *syp-1* mutants. Interestingly, both *him-3* and *htp-1* mutants have been proposed to be defective in inhibiting use of sister chromatids as DSBR partners (based on approximately normal kinetics of disappearance of RAD-51 foci despite lack of association between homologous partner chromosomes, suggesting the occurrence of sister chromatid-directed DSBR), whereas the barrier to sister-directed repair appears to be largely intact in *syp-1* and *syp-2* mutants (based on prolonged persistence of meiotic RAD-51 foci). It is striking that both (1) dependence of RAD-51 removal on association between homologs and (2) dependence of RAD-51 loading on RAD-50 are either retained or relieved in parallel under different mutant conditions, suggesting that these features are coordinately implemented as part of an integrated meiotic DSBR program. This in turn suggests that RAD-50 dependence of IR-induced RAD-51 focus formation can serve as a convenient surrogate for engagement of multiple aspects of the meiotic mode of DSBR.

How might HTP-1 and HIM-3 function to inhibit RAD-51 loading in the absence of RAD-50? One possibility is that these chromosome axis-associated proteins might generate a structural barrier that prevents alternative nuclease complexes from gaining access to DSBs generated within the constrained region. This type of mechanism would imply that DSBs may become associated with chromosome axis structures even if they are initially formed at distant positions within chromatin loops, as suggested previously by Blat et al. [51]. An alternative possibility is that the prolonged delay in RAD-51 accumulation at IR-induced breaks in *rad-50* mutants represents a checkpoint response, and that HIM-3 and HTP-1 play a role in the operation of this checkpoint. This model is appealing for several reasons. First, HTP-1 was previously proposed to function in checkpoint-like mechanisms that coordinate homolog recognition with SC assembly, in a manner analogous to the spindle assembly checkpoint [4]. Second, these proteins share an unusual conserved structural motif, the HORMA domain, with Mad2, a central player in the spindle assembly checkpoint [54]. Under this model, a DSB formed within the constrained region would generate a “wait resection” signal that inhibits progression of repair until a meiosis-specific recombination complex can be assembled; in a *rad-50* mutant, the conditions required to satisfy the checkpoint are not met, resulting in the observed delay in RAD-51 loading.

Our finding that germ cells revert to RAD-50 independence for RAD-51 loading in late prophase demonstrates the existence of a second developmentally programmed switch in the mode of DSBR. This switch occurs following a transient activation of MAP kinase that is required for progression from the mid-pachytene to late pachytene stage of meiotic prophase and is dependent on MAP kinase. Further, we showed that the switch occurs within the same time frame as loss of the capacity to convert IR-induced DSBs into interhomolog crossovers, suggesting that multiple aspects of the meiotic mode of DSBR are shut down simultaneously. We suggest that MAP kinase activation at the mid-late pachytene transition triggers a coordinate release from multiple constraints operating during earlier stages of meiotic prophase that together promote DSBR through formation of interhomolog crossovers.

This mid-late pachytene switch in DSBR mode occurs contemporaneously with a major remodeling of meiotic chromosome architecture. Whereas SC central region proteins (e.g., SYP-1) and meiotic chromosome axis proteins colocalize uniformly along the full lengths of synapsed homologs from early-mid pachytene, components of these structures undergo a dramatic relocalization beginning in late pachytene. Even prior to desynapsis, SYP-1 becomes concentrated to a localized domain on each homolog pair (in the vicinity of and distal to the single emerging chiasma), while a subset of chromosome axis components (including HTP-1) become concentrated to the reciprocal chromosomal domains ([12]; E. Martinez-Perez and A. Villeneuve, unpublished data).

In a recent analysis of synapsis-defective mutants [55], Smolikov et al. noted a correlation between chromosome organization within meiotic prophase nuclei (i.e., chromosomes clustered toward one side of the nucleus versus chromosomes dispersed about the nuclear periphery) and ability to remove SPO-11-dependent RAD-51 foci as an indicator of progression of DSBR. Based on their findings, they suggested a model in which chromosome clustering would inhibit some modes of DSBR, while chromosome dispersal might create an environment more permissive for multiple modes of DSBR. In the current analysis, we found that reversion to the RAD-50 independent mode of RAD-51 loading in *rad-50 syp-1* double mutants did not occur until chromosomes finally dispersed at the very end of the pachytene region, in keeping with the idea that persistent chromosome clustering may inhibit use of alternative modes of DSBR. However, our analysis also demonstrated that chromosome dispersal per se is not sufficient to confer a fully permissive DSBR environment.

### Conclusions

Our work provides insight into the mechanisms that allow germ cells to generate recombination-based linkages that promote segregation of homologous chromosomes at meiosis while at the same time safeguarding the integrity of the genome. We show that *C. elegans* germ cells engage a specialized mode of DSBR at the onset of meiotic prophase, characterized by dependence on RAD-50 for rapid accumulation of RAD-51 at DSBs and by competence to convert DNA breaks into interhomolog crossovers. This requirement for RAD-50 is imposed, at least in part, by meiosis-specific differentiation of chromosome axis structures. Our data further suggest a model in which features of chromosome structure conferred by the meiotic cohesin component REC-8 may limit the activity of SPO-11 in generating meiotic DSBs. Finally, we show that germ cells undergo a second developmentally programmed switch in DSBR mode at the mid-late pachytene transition, characterized by reversion to a RAD-50 independent mode of RAD-51 loading and loss of competence to generate interhomolog crossovers.

Observations from several different experimental systems suggest that a capacity to switch from a highly specialized meiotic DSBR mode to a less constrained DSBR mode during prophase progression may be a widespread feature of meiotic programs. For example, Mehrotra and McKim [47] recently reported that formation of γ-His2Av foci in response to X-ray-induced DSBs occurs much more slowly in the early pachytene stage than in the late pachytene stage during Drosophila female meiosis, even in repair-proficient germ cells. The authors propose that this may reflect repression of the normal DSB response during early meiotic prophase, followed by alleviation of this repression at the transition to the late pachytene stage. Further, during mouse spermatocyte meiosis, disappearance of DSB-associated markers from heterosynapsed chromosome regions occurs in a later time window than disappearance of markers from correctly synapsed chromosomes [53], likewise suggesting a change in the rules governing DSB repair during prophase progression. We suggest that a late prophase switch to a less constrained DSBR environment serves as fail-safe mechanism for safeguarding the genome by providing an opportunity to restore chromosome integrity prior to chromosome segregation.

## Materials and Methods

### Genetics.

Except where specified, all C. elegans strains were cultured at 20 °C under standard conditions [56]. The following mutations and chromosome rearrangements were used: *Chromosome III: unc-79(e1068), mpk-1(ga111ts); Chromosome IV: dpy-13(e184) , htp-1 (gk174), him-3(gk149), spo-11(ok79, me44), rad-51(lg8701), rec-8(ok978), dpy-4(e1166); Chromosome V: mre-11(ok179), rad-50 (ok197), syp-1(me17) Balancers: nT1 IV;V. nT1[ unc-?(n754) let-? qIs50] IV; nT1 V. nT1 IV; nT1[qIs51] V*

The following balanced heterozygous stocks were generated for this analysis:

AV158 *+/ nT1 [unc-?(n754) let-? qIs50] IV; rad-50 (ok197)/ nT1 V*

AV270 *spo-11(ok79)/ nT1[ unc-?(n754) let-? qIs50] IV; rad-50 (ok197)/ nT1 V*

AV457 *+/ nT1 IV; rad-50 (ok197) syp-1(me17)/ nT1 [qIs51]V*

AV451 *him-3(gk149)/nT1[ unc-?(n754) let-? qIs50] IV; rad-50 (ok197)/ nT1 [qIs51] V*

AV443 *htp-1 (gk174)/nT1[ unc-?(n754) let-? qIs50] IV; rad-50 (ok197)/ nT1 [qIs51] V*

AV414 *dpy-13(e184) rad-51(lg8701)/nT1[ unc-?(n754) let-? qIs50] IV; rad-50 (ok197)/ nT1 V*

AV417 *rad-51(lg8701)/nT1[unc-?(n754) let-? qIs50] IV; rad-50 (ok197)/ nT1 V*

AV423 *rec-8(ok978)/nT1[unc-?(n754) let-? qIs50] IV; rad-50 (ok197)/ nT1 [qIs51] V*

AV468 *rec-8(ok978) dpy-4(e1166)/nT1 IV; rad-50 (ok197)/ nT1 [qIs51] V*

AV469 *spo-11(me44) rec-8(ok978) dpy-4(e1166)/nT1 IV; rad-50 (ok197)/ nT1 [qIs51]V*

AV462 *unc-79(e1068) mpk-1(ga111ts) III; +/ nT1 [unc-?(n754) let-? qIs50] IV; rad-50 (ok197)/ nT1 V*

For all experiments with meiotic mutants, homozygous mutant worms were derived from balanced heterozygous parents either by selecting progeny worms lacking a dominant marker (Unc and/ or GFP) associated with the balancer chromosome or by selecting worms homozygous for a *cis*-linked recessive marker.

To evaluate acquisition of IR-induced RAD-51 foci in *unc-79 mpk-1(ga111ts); rad-50* hermaphrodites at non-permissive temperature, we shifted *unc-79 mpk-1(ga111ts); rad-50 (ok197)/ nT1 [ unc-?(n754) let-? qIs50] IV;V* gravid adults from 15 °C to 26 °C, allowed them to produce self-progeny, and selected non-GFP progeny at the L4 stage. At 20 h post L4, worms were exposed to IR and subsequently processed for IF as described below.

The *rad-50(ok197)* deletion was isolated from unbalanced heterozygotes obtained from the *C. elegans* Gene Knockout Consortium. Sequencing of the deletion fragment amplified by PCR from *rad-50(ok197)* homozygotes revealed a deletion of 1,571 bp, from coordinates 12246298 to 12247814 on chromosome V. The sequences flanking the deletion are: gaatttcactttacttagtc, tgaaattatttagcactgcc.

The *spo-11(me44)* allele contains a G to A transition at coordinate 11125782 of chromosome IV, the last coding base of exon 6. Although this represents a change in the splice donor sequence, this change is not predicted to interfere with splicing and is instead predicted to act as a missense mutation, changing a highly conserved Glycine to an Aspartic acid at residue 290 of the 425 amino acid protein. The affected residue is in the middle of the TOPRIM_TopoIIB_SPO cd00223 motif (at the junction between the TOPRIM domain and the dimer interface) and is conserved as a Glycine in all SPO11 proteins for which functional data are available [2] as well as in 92% of 51 aligned sequences defining the motif in CDD. Previous functional characterization indicates that *spo-11(me44)* behaves as a severe loss-of-function or null allele in meiotic recombination assays [57].

*rec-8(ok978)* is an insertion/deletion allele in which 1,577 bp of the *rec-8* gene are deleted and replaced by an insertion of 371 bp. Depending on the outcome of RNA processing, the altered gene may have the capacity to encode for a truncated product comprising less than half of the 781 amino acids present in the wild-type protein. Comparison with previously-reported *rec-8(RNAi)* and *rec-8(cosuppression)* phenotypes [3,21,35] suggests that *rec-8(ok978)* severely reduces or eliminates gene function.

### Cytological analysis.

Except where noted below, fixation, DAPI-staining, immunostaining and acquisition and processing of images using the Deltavision deconvolution microscopy system was carried out as in [4], using gonads dissected from 18–20 h post-L4 adults. The following primary antibodies were used at the indicated dilutions: guinea pig α-SYP-1 (1:200) [11], rabbit α-RAD-51 (1:50) [3], rabbit α-HIM-3 (1:200) [10], chicken α-HTP-3 (1:500) [19], guinea pig α-HIM-8 (1:500) [20]. Secondary antibodies used were: Alexa 488 α-rabbit, Alexa 555 α-guinea pig, Alexa 555 α-chicken. For most double-labeling experiments, primary antibody incubations were performed simultaneously, followed by simultaneous incubation with secondary antibodies. For SYP-1 and RAD-51 double staining, slides were incubated sequentially with α-RAD-51 primary, α-rabbit secondary, α-SYP-1 primary, α-guinea pig secondary. Images were collected in z-series representing a longitudinal bisection of each gonad arm; images shown are projections through data stacks encompassing whole nuclei.

For most experiments assessing formation of RAD-51 foci following IR treatment, 20 h post-L4 worms were exposed to 1 krad of γ-irradiation from a ^137^Cs source; gonads were dissected and fixed for IF at 1 h post irradiation. The 1 krad dose used in these experiments is 5–7-fold lower than the doses typically used in most prior analyses of DSBR in *C. elegans* germ cells (e.g., [4,18,39,40]). This dose was used because it was sufficient to elicit abundant RAD-51 foci in both wild-type and *spo-11* mutant germ lines and to restore chiasma formation in a *spo-11* mutant [22], but does not lead to the poorly condensed, aggregated and/or fragmented appearance of chromosomes observed in diakinesis-stage oocytes in *mre-11* or *rad-50* mutants following a 5 krad exposure ([18] and unpublished data). For Figure S3, a 5 krad dose was used; for Figures 5, S5, and S6, germ lines were fixed at the indicated times following irradiation at the 1 krad dose.

For Figure 2B and 2C, gonads were dissected from 21 h post-L4 adults; slides were transferred to 100% methanol at −20 °C for 30–60 s, then post-fixed with 4% formaldehyde in 1× PBS, 80 mM HEPES, pH 6.9, 1.6 mM MgSO_4_ for 30 min. Slides were washed in PBST and held at 4 °C until post-fixing was complete for all slides. Slides were blocked with 0.5% BSA for 1 h, then incubated with RAD-51 antibody (1:100 dilution in 50 μL PBST for 2 h at room temperature, then overnight at 4°C. Slides were washed 3× in PBST for 15 min; Cy3-labelled secondary antibody (Jackson Laboratories) was applied at a dilution of 1:200 in PBST and slides were incubated as for primary antibody. Slides were washed 3× in PBST for 15 min then stained with DAPI, mounted, and imaged, as in [11]. Quantitation of RAD-51 foci in zones distributed along the distal-proximal gonad axis was performed as in [3]. Correspondence of stages indicated on Figure 2B to the zones used in Colaiacovo et al. are as follows: “premeiotic (pm)”, zones 1 and 2; “transition zone (tz)”, zone 3; “early-mid pachytene (e/m pt)”, zones 4 and 5; “late pachytene (l pt)”, zone 6 (Note that zone 6 in [3] corresponds to zone 7 in several subsequent studies, e.g., [4]). Numbers of nuclei scored were as follows: Wild-type: pm, 424; tz, 191; e/m pt, 285; l pt, 91. *rad-50:* pm, 248; tz, 78; e/m pt, 134; l pt, 54. *spo-11*: pm, 565; tz,168; e/m pt, 416; l pt,118. *spo-1; rad-50:* pm, 292; tz, 149; e/m pt, 261; l pt, 89.

For Figure 4C, double labeling with α-RAD-51 and mouse monoclonal antibody M8159 (Anti-MAP Kinase, Activated [Diphosphorylated ERK-1&2] [Sigma], used at 1:1,000 dilution) was performed following the method of [58], with modifications (Y Sasagawa, personal communication). Gonads dissected from 20 h post-L4 worms were fixed in 2% paraformaldehyde for 1 h at room temperature, then post-fixed for 10 min with dimethylformamide at −20 °C. Slides were washed with 1× PBST three times for 10 min and blocked with 3% BSA, 2mM MgCl_2_, 0.1% Tween-20 for 20 min. Slides were incubated with primary antibodies in 1× PBS, 0.1%BSA, 0.5%Triton X-100, 0.05% sodium azide,1mM EDTA overnight in a humid chamber at 4 °C. Slides were washed as above and incubated with secondary antibodies (Alexa 488 α-rabbit and Alexa 555 α-mouse) for 2 h at 25 °C.

### Quantitation of achiasmate chromosomes in ooctye nuclei.

For quantitation of achiasmate chromosomes in the *rad-50(ok197*) mutant, whole worms were fixed with Carnoy's fixative at 48 h post L4 and stained with DAPI as in [59]. For experiments assessing chiasma formation in *spo-11(ok79)* worms following exposure to ionizing radiation, whole worms were fixed in ethanol and stained with DAPI as in [60]. Twenty h post-L4 adult worms were exposed to 1 krad of γ-irradiation from a ^137^Cs source, and cohorts were fixed 12, 14, 16, or 18 h later; unirradiated controls were fixed at the 18-h time point (38 h post-L4). Since individual univalents or bivalents in some oocyte nuclei lie too close to each other to be resolved unambiguously, these assays tend to underestimate the frequency of achiasmate chromosomes. Although the dose used in these experiments was 5-fold lower than that used in previous reports (5 krad), sufficient breaks were generated to restore chiasma formation for the full complement of chromosomes at high frequency.

## Supporting Information

Figure S1 Successful Pairing of Chromosome V in the *rad-50* Mutant Pachytene nuclei from a *rad-50* mutant germ line hybridized with a FISH probe (orange) targeting the 5S locus on chromosome V. A single hybridization signal or a pair of closely-spaced signals is present in each nucleus, indicative of intimate pairing of homologous chromosomes. Scale bar = 2 μm.
(1.5 MB TIF)

Figure S2 Both *rad-50* and *mre-11* Mutants Are Defective in Rapid Accumulation of RAD-51 at IR-Induced Breaks from Meiotic Prophase Onset through the Mid-Pachytene Stage Germ lines from wild type (WT), *rad-50*, and *mre-11* hermaphrodites fixed 1 h after exposure to 1 krad γ-irradiation and stained with DAPI and RAD-51 antibody. RAD-51 foci are detected in germ cell nuclei throughout the wild-type gonad, from the proliferating nuclei at the distal tip (left) through nuclei at the diplotene stage of meiotic prophase (right). In the *rad-50* and *mre-11* mutants, in contrast, RAD51 foci are absent in most nuclei in the central portion of the gonad, indicated by the bracket, from the onset of meiotic prophase through the mid-pachytene/late pachytene transition (see Figure 4). Scale bar = 5μm.
(9.6 MB TIF)

Figure S3 Response of Germ Cells to 5 krad Dose of γ-Irradiation Germ lines from *rad-50* and *htp-1; rad-50* hermaphrodites fixed 1 h after exposure to 5 krad γ-irradiation and stained with DAPI and RAD-51 antibody. As in experiments using 1 krad IR treatment, strong inhibition of IR-induced RAD-51 foci is observed in the central portion of the *rad-50* mutant germ line (indicated by bracket); moreover, because RAD-51 foci in premeiotic and late prophase germ cells are more abundant following this higher dose, the contrast between these regions and the inhibited zone is even more pronounced than at the lower dose. Abundant RAD-51 foci are present throughout most of the central portion of the *htp-1; rad-50* germ line, indicating that inhibition of IR-induced RAD-51 foci is abrogated in this double mutant. The *htp-1; rad-50* germ line does retain a reduced domain (indicated by bracket) in which most nuclei lack RAD-51 foci, indicating that inhibition of IR-induced RAD-51 focus formation is not completely eliminated. Scale bar = 5 μm.
(5.8 MB TIF)

Figure S4 Reversion to the RAD-50-Dependent Mode of RAD-51 Loading Requires MPK-1 (MAP kinase) Germ lines from *mpk-1(ga111ts)* (control) and *mpk-1(ga111ts); rad-50* hermaphrodites raised at 26 °C, fixed 1 h after exposure to 1 krad γ-irradiation and stained with DAPI and RAD-51 antibody. Abundant IR-induced RAD-51 foci are seen in the premeiotic region of the *mpk-1; rad-50* germ line, followed by a “dark” region indicative of switching to the RAD-50-dependent mode of RAD-51 loading at the onset of meiotic prophase. However, meiotic prophase does not progress beyond the pachytene stage, and there is no second zone with abundant IR-induced RAD-51 foci. Failure to detect reversion to the RAD-50-independent mode of RAD-51 loading does not imply a direct regulation of RAD-51 by MAP kinase, but rather indicates that developmental progression to the late pachytene stage (which is dependent on MAP kinase) is required for the switch in DSBR mode. Scale bar = 20 μm.
(2.4 MB TIF)

Figure S5 Persistence of Preassembled RAD-51 Foci Following Entry into Meiotic Prophase Distal portion of a germ line from a *spo-11; rad-50* mutant worm, extending from the distal tip to mid-pachytene region, fixed 12 h after exposure to 1 krad γ-irradiation and stained with HTP-3 and RAD-51 antibodies. The arrows indicate the region containing nuclei with concentrated HTP-3 signals indicative of meiotic prophase chromosome structures. In contrast to the 1 h time point, where abundant bright RAD-51 foci and meiotic HTP-3 signals are mutually exclusive (Figure 4), abundant bright RAD-51 foci are present in transition zone and early pachytene nuclei (which contain extensive meiotic HTP-3 signals) at this 12 h time point. Based on recent temporal analyses of progression through meiotic prophase ([26]; S. Mlynarczyk-Evans and A. Villeneuve, unpublished data), we infer that these nuclei in early meiotic prophase at the 12 h time point had been in the premeiotic region at the time of irradiation. In more proximal positions (right), many meiotic prophase nuclei exhibit smaller, more discrete RAD-51 foci, apparently reflecting delayed accumulation of RAD-51 at break sites; nuclei in this region were already in the constrained region at the time of irradiation. Scale bar = 5 μm.
(6.6 MB TIF)

Figure S6 Comparisons of RAD-51 Foci at 1 h and 12 h Post-Irradiation Time Points Images shown are portions of *spo-11* and *spo-11; rad-50* germ lines from (A) the premeiotic region, (B) the mid-pachytene region, and (C) the late pachytene/diplotene/early diakinesis region, fixed at 1 or 12 h after exposure to 1 krad γ-irradiation and stained with DAPI and RAD-51 antibody. In contrast to all other pairs of panels, which show a reduction in the number of foci per nucleus and/or the fraction of nuclei with foci between the 1 and 12 h time points, there was a clear increase in the incidence of foci in the mid-pachytene region of the *spo-11; rad-50* mutant (B). As nuclei in the mid-pachytene region at the 12 h time point were already in meiotic prophase at the time of irradiation, this rise in the incidence of foci reflects delayed accumulation of RAD-51, presumably at break sites. Whereas a detailed analysis of the kinetics of disappearance of RAD-51 foci will be presented elsewhere, we highlight here several additional point illustrated by this figure: (A) Whereas abundant RAD-51 foci are detected in nuclei in the premeiotic region (for both *spo-11* and *spo-11; rad-50*) at the 1 h time point, at the 12 h time point the premeiotic region contains two distinct classes of nuclei—one with bright RAD-51 signals and one lacking RAD-51 foci. These two classes are already evident at 5 h post irradiation (unpublished data). We suggest that these two classes may reflect distinct responses in nuclei that were in different phases of the cell cycle at the time of irradiation; we hypothesize that although germ cell nuclei in all phases of the mitotic cycle are competent to load RAD-51, only a subset of nuclei are competent to complete repair (and presumably to resume cycling). Further, the persistent RAD-51 foci suggest the formation of unproductive RAD-51-containing complexes in the other class of nuclei. The two classes of nuclei are found in the premeiotic regions of both *spo-11* and *spo-11; rad-50* germ lines (see also Figure 5), suggesting that some RAD-50-independent repair may occur in proliferating germ cells. Persistent RAD-51 foci do not represent a barrier to meiotic prophase entry, as nuclei with persistent bright foci are observed in the transition zone and early pachytene regions of both *spo-11* and *spo-11; rad-50* germ lines at the 12 h time point (Figure S5; unpublished data), reflecting movement and developmental progression of nuclei that were in the premeiotic region at the time of irradiation. (B) RAD-51 foci in the mid-pachytene region decline in abundance between the 1 and 12 h time points in the *spo-11* mutant, but most nuclei retain at least one or a few RAD-51 foci at the 12 h time point; this suggests that within the constrained region, repair is ongoing but is not yet complete by 12 h after irradiation. (C) Nuclei in the diplotene and diakinesis stages at the 12 h time point are inferred to correspond to nuclei that were in the late pachytene region at the time of irradiation, and nuclei in the late pachytene region at the 12 h time point are inferred to correspond to nuclei that were in the mid-pachytene region at the time of irradiation. In the 12 h *spo-11* germ line, foci are reduced in abundance in late pachytene and diplotene nuclei and are absent by early diakinesis, suggesting that repair may be completed by the time nuclei reach the diakinesis stage. In the 12 h *spo-11; rad-50* germ line, there is a decline in foci in late diplotene and early diakinesis nuclei, but foci are not completely eliminated by diakinesis; this suggests that some RAD-50-independent repair may occur in late prophase germ cells, but not all lesions are eliminated. Scale bars in A and B = 5 μm; scale bar in C = 7.5 μm.
(14.7 MB TIF)

## Abbreviations

CO: crossover
DAPI - 4′: 6-diamidino-2-phenylindole
DSB: double-strand break
DSBR: double-strand break repair
FISH: fluorescence in situ hybridization
IF: immunofluorescence
IR: ionizing radiation
SC: synaptonemal complex.
